# Comparative Effectiveness of Insulin Versus Oral Hypoglycemic Agents in Diabetic Patients With Acute Stroke: A Retrospective Cross‐Sectional Analysis of Treatment Outcomes and Influencing Factors

**DOI:** 10.1002/hsr2.71598

**Published:** 2025-12-09

**Authors:** Mohsen Salehi, Mehdi Maghbooli, Mohsen Bagheri, Seyed Nariman Tavakoli Sany, Melina Arfaei

**Affiliations:** ^1^ Faculty of Medicine Zanjan University of Medical Sciences Zanjan Iran; ^2^ Department of Neurology, Faculty of Medicine Zanjan University of Medical Sciences Zanjan Iran; ^3^ Student Research Committee, Department of Neurology, Faculty of Medicine Zanjan University of Medical Sciences Zanjan Iran; ^4^ Student Research Committee, Faculty of Medicine Zanjan University of Medical Sciences Zanjan Iran

**Keywords:** diabetes, insulin, oral hyperglycemic drug, stroke

## Abstract

**Background and Aims:**

Diabetes is a disease that presents numerous challenges for both patients and physicians. Researching the factors associated with this condition is essential in today's medical studies. This study aims to determine the type of therapeutic regimen—either insulin or oral hypoglycemic agents—used in diabetic patients who have experienced an acute stroke.

**Methods:**

This descriptive‐comparative study focused on diabetic patients who were referred to Valiasr Hospital in Zanjan in 2021 due to an acute stroke. Out of the initial statistical population, 248 individuals were evaluated using a demographic questionnaire and a checklist. After excluding incomplete questionnaires, a total of 176 participants were included in the analysis. The data were entered into SPSS v.26 for statistical analysis, which involved both descriptive methods (mean, standard deviation, frequency, and percentage) and inferential methods (*χ* ^2^ test).

**Results:**

The findings of this study indicate that patients' conditions—assessed through MRS, MRS post‐discharge, NIHSS, atrial fibrillation rhythm, sex, stroke location, and type of stroke—were not significantly associated with the treatment regimens, whether insulin or oral medications. However, as age increased, the amount of insulin used compared to oral medications showed a significant rise.

**Conclusion:**

Our findings indicated that diabetic patients who received insulin had a similar risk of acute stroke compared to those treated with oral medications. However, research shows that diet therapy plays a significant role in the management and progression of diabetes. Therefore, further studies in this area are needed to address the potential negative consequences of ischemia.

## 1 Introduction

Stroke is an acute neurological injury that occurs when blood flow to a specific area of the brain is interrupted. There are two main types of strokes: ischemic stroke, which is caused by a blockage in a blood vessel, and hemorrhagic stroke, which occurs when a blood vessel in the brain ruptures. As the population ages, the prevalence and burden of disability from stroke are expected to increase. In addition to age, the primary risk factors for stroke include hypertension, diabetes mellitus (DM), physical inactivity, smoking, and hyperlipidemia (HLP) [1, 2].

Diabetes mellitus is a recognized risk factor for acute stroke. Furthermore, many studies have shown a connection between acute hyperglycemia and larger infarct volumes, as well as poorer functional and rehabilitation outcomes in stroke patients [3, 4]. Multiple mechanisms through which diabetes mellitus causes stroke have been identified. These mechanisms include an increased risk of premature death, arterial stiffness, vascular endothelial dysfunction, and thickening of the basal capillary membrane [5].

There is evidence that intensive treatment of hyperglycemia reduces the risk of microvascular complications in patients with diabetes mellitus. However, it remains inconclusive how beneficial this intensive treatment is in terms of stroke incidence compared to conventional glucose control methods. Additionally, insulin resistance (IR) plays a role in endothelial dysfunction and atherosclerosis [6, 7]. Many studies indicate that insulin resistance increases the risk of stroke, its recurrence, and negatively affects cognitive function [8]. Insulin‐treated diabetic patients have a higher risk of stroke compared to those treated with oral medication or nondiabetic patients. Patients on oral medication also exhibit increased stroke risk relative to nondiabetic patients [9].

Patients who require insulin treatment often experience worse outcomes, suggesting that their disease may be more advanced and that they have a higher risk of complications. While insulin can help prevent myocardial infarction (MI), it may also increase the risk of stroke in individuals with diabetes mellitus (DM). Therefore, effective management of diabetes is crucial. In older adults, the decision to use insulin for controlling blood glucose levels should be made with caution. Oral medications that can mitigate the negative effects of insulin while maintaining its ability to regulate blood sugar would be especially valuable in treatment [10].

The aim of this investigation is to explore the various factors that influence the decision‐making process when choosing between insulin and oral hypoglycemic drugs for diabetic patients who have suffered an acute stroke. By thoroughly examining these factors, we hope to provide insights that can enhance clinical protocols and inform optimal treatment approaches, ultimately leading to improved patient outcomes in this vulnerable population.

## 2 Methods

### 2.1 Study Design

This retrospective cross‐sectional study included all adult diabetic patients (aged ≥ 18 years) admitted to Valiasr Hospital, Zanjan, Iran, between January 1, 2020 and December 31, 2020, with a radiologically confirmed diagnosis of stroke (ischemic, hemorrhagic, or transient ischemic attack [TIA]). A complete census sampling method was employed due to the limited cohort size.

Inclusion criteria

- Diagnosis of type 1 or 2 diabetes mellitus (defined by ADA criteria: HbA1c ≥ 6.5% or documented use of antidiabetic medications).
- Acute stroke confirmed by neuroimaging (CT or MRI).
- Availability of complete medical records, including discharge summaries and follow‐up data.

Exclusion criteria

- History of thrombophilia (e.g., hereditary thrombophilia, active malignancy‐associated thrombosis).
- Terminal illness (e.g., metastatic cancer, end‐stage organ failure).
- Incomplete clinical or imaging data.

### 2.2 Data Collection

Upon receiving approval for the research proposal, we conducted a comprehensive review of existing literature in the diabetes and stroke management field. We extracted relevant questions from the background information and patient files, filling in any missing details by consulting with the patients. Subsequently, we collaborated with experts in the field to develop a custom questionnaire. The next step involved data collection in the field using a checklist encompassing demographic variables such as age and gender, along with the clinical characteristics of patients, including the type and location of stroke, diabetes control methods, and details of medications and dosages.

### 2.3 Variables

#### 2.3.1 Primary Outcomes

1. Stroke severity

- Assessed using the National Institutes of Health Stroke Scale (NIHSS) at admission (range: 0–42; higher scores indicate greater impairment).
- Categorized as:
  ◦ Mild (0–5),
  ◦ Moderate (6–14),
  ◦ Severe (≥ 15).

2. Functional disability

- Measured using the Modified Rankin Scale (mRS) at two time points:
  ◦ At discharge
  ◦ Months post‐discharge (via clinic visit or structured telephone interview).

- Graded as:
  ◦ 0: No symptoms.
  ◦ 1–2: Mild disability (independent in daily activities).
  ◦ 3–5: Moderate to severe disability (dependent on assistance).
  ◦ 6: Death.

#### 2.3.2 Secondary Outcomes

1. Hospital length of stay (LOS)

- Categorized as:
  ◦ Short ( ≤ 1 day)
  ◦ Intermediate (2–5 days)
  ◦ Prolonged ( ≥ 6 days).

2. Stroke characteristics
  - Type: Ischemic, hemorrhagic, or TIA.
  - Location: Anterior circulation (MCA, ACA), posterior circulation (POS), or undetermined.

3. Glycemic control and diabetes management
  - HbA1c level (categorized as < 7%, 7%–9%, > 9%).
  - Antidiabetic therapy: Insulin, oral agents (metformin, sulfonylureas), or newer agents (SGLT2 inhibitors, GLP‐1 agonists).

4. Comorbidities and risk factors
  - Hypertension (BP ≥ 140/90 mmHg or documented diagnosis).
  - Dyslipidemia (LDL > 100 mg/dL or lipid‐lowering therapy).
  - Atrial fibrillation (ECG‐confirmed).
  - Chronic kidney disease (eGFR < 60 mL/min/1.73 m²).

5. Acute stroke interventions
  - Intravenous thrombolysis (rtPA) (yes/no).
  - Door‐to‐needle time (minutes).

### 2.4 Data Analysis

Data were analyzed using SPSS version 26 (IBM Corp., Armonk, NY). Continuous variables were reported as mean ± standard deviation (SD) or median (interquartile range, IQR), depending on normality (assessed via Shapiro–Wilk test). Categorical variables were presented as frequencies (percentages).

#### 2.4.1 Inferential Analysis

- Group comparisons:
  ◦ *χ* ^2^ or Fisher's exact tests for categorical variables.
  ◦ Independent *t*‐tests or Mann–Whitney *U* tests for continuous variables.
- Predictors of poor outcome (mRS ≥ 3):
  ◦ Multivariable logistic regression adjusted for age, sex, NIHSS score, and comorbidities.

- Missing data:
  ◦ Excluded if > 10% missing per variable.
  ◦ Sensitivity analysis performed using multiple imputation.

A two‐tailed *p* value < 0.05 was considered statistically significant.

### 2.5 Ethical Consideration

− The study was conducted after obtaining approval from the university's ethics committee with the assigned code IR.ZUMS.REC.1399.274.

− No coercion was imposed, and no interference in patient treatment was made, with no additional cost incurred.
− Patient information remained confidential, with each individual assigned a code and patients' names not disclosed.

− Participants were assured that the questionnaires would be solely used by the researcher and not for any other purpose.
− The content of the study was not contradictory to the cultural norms.
− The names of all research team members were mentioned in the outputs of the thesis.

## 3 Result

### 3.1 Descriptive Data

**Figure 1:**
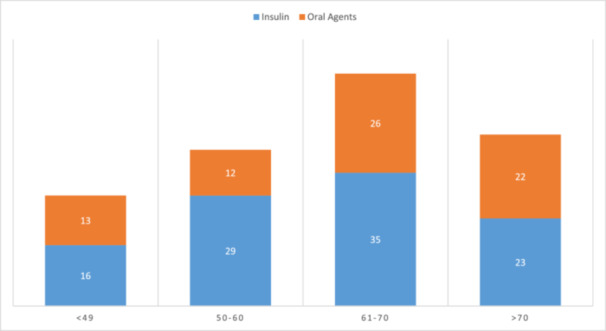
Comparing the type of treatment regimen of study participants according to age with the *χ* ^2^ test.

The study included all diabetic patients who visited Vali‐Asr Hospital at Zanjan University of Medical Sciences in 2020 and were diagnosed with a stroke based on CT or MRI scans. A total of 906 stroke patients were screened, of whom 262 (28.9%) had diabetes. After excluding 86 patients (32.8%) due to incomplete records or meeting exclusion criteria, 176 patients were included in the final analysis (Figure 1). The cohort comprised 100 males (56.8%) and 76 females (43.2%), with a mean age of 66.2 ± 13.0 years (range: 31–98 years). Patients aged > 71 years represented the largest subgroup (45.6%). Data were collected from medical records and supplemented with patient interviews to address missing information. A checklist captured demographic variables (age, gender) and clinical characteristics (stroke type, diabetes medication, comorbidities, etc.)

Diabetes was managed with injectable insulin in 94 patients (53.4%) and oral medications in 82 (46.6%). Among oral medication participants, 111 were prescribed metformin, 19 sulfonylurea, 1 pioglitazone, 2 sodium‐glucose transporter (SGLT) inhibitors, and 2 GLP‐1 agonists, indicating some patients used multiple oral drugs.

Participants had a mean hospital stay of 7.53 days (SD = 11.55 days, range 1–132 days). Regarding time to arrival index (TAI), 105 patients (59.7%) arrived after 4.5 h, while 52 (29.5%) arrived within 4.5 h; TAI data were missing for 19 patients.

Smoking was reported by 24 patients (13.6%), with 152 (86.4%) being non‐smokers. Comorbidities were prevalent: hypertension (149, 84.7%), ischemic heart disease (49, 27.8%), prior cerebral vascular accident (28, 15.9%), dyslipidemia (23, 13.1%), kidney disease (12, 6.8%), chronic heart failure (12, 6.8%), atrial fibrillation (7, 4.0%), malignancy (2, 1.1%), valvular disease (2, 1.1%), and liver disease (2, 1.1%).

### 3.2 Findings

Figure 1: Comparing the type of treatment regimen of study participants according to age with *χ*2 test.

Based on the data, a significant association was observed between insulin use and increasing age (*β* = 8.6 years; 95% CI: 0.8–16.4; *p* = 0.048, linear regression), accounting for 12% of the variance (*R*² = 0.12). No sex‐based differences in treatment allocation were detected (46.8% females on insulin vs. 39.0% on oral agents; *p* = 0.18, *χ*2).

Based on the data presented in the Table 1, it was determined that there is no statistically significant correlation between the NIHSS and MRS scores and the type of treatment regimen in diabetic patients with acute stroke.

**Table 1:** Distribution of NIHSS and MRS scores of study participants.

Outcome	Time point	Score	Oral agents (*n* = 82)	Insulin (*n* = 94)	*p* value
NIHSS	Discharge	0	0	0	0.32
		1–4	18	17	
		5–15	33	38	
		16–20	21	22	
		21–42	18	9	
MRS	Discharge	0	13	12	0.87
		1	7	7	
		2	24	29	
		3	13	7	
		4	12	12	
		5	9	15	
		6	4	12	
	3 months follow‐up	0	24	19	0.15
		1	7	14	
		2	13	11	
		3	6	11	
		4	15	12	
		5	3	7	
		6	10	24	

Based on the Table 2, no significant association was observed between stroke type (ischemic/hemorrhagic/TIA) or location (anterior/posterior circulation) and antidiabetic treatment regimen (all *p*> 0.05, Fisher's exact test).

**Table 2:** Distribution of stroke type and location of study participants.

Characteristic		Oral agents	Insulin	Total number	*p* value
Stroke Type	Ischemic	79	87	166	0.31
	Hemorrhagic	2	5	7	0.45
	TIA	1	2	3	1.00
Stroke location	POS	1	1	2	1
	MCA	71	76	147	0.3
	ACA	6	3	9	0.31
	Undetermined	2	10	12	0.03

The study's findings revealed that administration of rtPA did not differ by antidiabetic regimen (17.0% insulin users vs. 14.6% oral agent users; *p* = 0.54, *χ* ^2^ test).

Our result showed patients with abnormal atrial fibrillation rhythms showed no significant preference for insulin (90/168, 53.6%) versus oral agents (78/168, 46.4%) compared to those with normal rhythms (5/8 insulin, 3/8 oral agents; *p* = 0.55, Fisher's exact test).

In our sample, 82% of patients (47 individuals) were using oral hypoglycemic drugs. At the time of data collection (December 31, 2023), 18% of these patients (8 individuals) were recorded as deceased. In contrast, patients using insulin had a mortality rate of 30%. These differences were not statistically significant and did not influence the parameters examined in this study (*p* = 0.3).

## 4 Discussion

We found that in diabetic patients with acute stroke, older age was independently associated with higher insulin use (adjusted OR per decade: 1.8, 95% CI: 1.3–2.5; *p* = 0.001), while no significant associations were observed with gender, stroke type/location, atrial fibrillation, thrombolysis, or functional outcomes (NIHSS/MRS scores at discharge/3 months; all *p* > 0.05). These findings suggest that age‐related physiological changes (e.g., insulin resistance, β‐cell dysfunction) may drive treatment selection more strongly than acute stroke characteristics.

In line with our findings, a study by Rubeaan et al. aimed to investigate risk factors associated with stroke in diabetic patients. Poor diabetes control, diabetes‐related complications, and smoking were associated with an increased risk of stroke. Furthermore, regression analysis indicated that being over 45 years old, male gender, hypertension, coronary artery disease, having diabetes for more than 10 years, insulin use, and hyperlipidemia were independently significant risk factors for stroke [11]. Similarly, Mozhgan et al. also demonstrated that aging exacerbates diabetes complications (e.g., neuropathy, retinopathy), necessitating insulin therapy in 62% of patients > 65 years versus 28% of younger patients (*p* < 0.001) [12]. Together, these findings underscore age as a critical determinant of both stroke risk and treatment intensity in diabetes.

This age‐dependent shift toward insulin therapy likely reflects sarcopenic obesity—a hallmark of aging characterized by muscle loss and visceral fat accumulation [13]. Adipose tissue expansion promotes systemic inflammation and insulin resistance [14], necessitating higher exogenous insulin doses to maintain glycemic control. However, chronic insulin administration may further reduce hepatic insulin clearance [15], and promote adipogenesis, creating a vicious cycle of weight gain and worsening insulin resistance (OR: 1.4 per 5‐year age increase; 95% CI: 1.1–1.8). In stroke patients, this metabolic dysregulation is particularly concerning, as both hyperinsulinemia and obesity are linked to endothelial dysfunction and recurrent stroke risk [16]. Thus, age‐related physiological changes may explain the therapeutic reliance on insulin despite its potential adverse effects.

While our study and Kaviani et al. [12] found no sex‐based differences in diabetes treatment choice (insulin vs. oral agents: men 48% vs. women 52%; *p* = 0.34), the broader literature reveals important contextual factors. Although men generally have higher stroke incidence (OR: 1.3, 95% CI: 1.1–1.5) [17], this gap narrows in diabetic populations (OR: 1.1, 95% CI: 0.9–1.3), possibly due to women's elevated diabetes risk from hormonal changes (e.g., menopause‐associated adiposity redistribution) [18]. Notably, women with diabetes show 18% higher medication adherence rates in meta‐analyses [19], which may compensate for their greater metabolic vulnerability. These converging factors—comparable treatment needs but distinct biological risks—explain why sex does not significantly predict regimen selection in our cohort.

Insulin and insulin‐sensitizing medications demonstrate antithrombotic properties that may confer stroke protection in diabetes. Metformin reduces platelet aggregation by 30%–40% through AMPK‐dependent pathways [20], while insulin decreases fibrinogen levels by 15%–20% and improves endothelial nitric oxide bioavailability [21]. These effects collectively lower thrombotic risk, particularly for lacunar strokes (OR: 0.7, 95% CI: 0.5–0.9) [22]. Paradoxically, diabetic patients still form more compact fibrin clots (25% higher fiber density by electron microscopy) [23] with reduced lysability, explaining their residual stroke risk despite treatment. This duality underscores the need for combined approaches targeting both metabolic control and hemostatic pathways.

Chen et al. systematically analyzed the diabetes‐stroke nexus, demonstrating that each 1 mmol/L increase in fasting glucose elevates stroke risk by 11% (95% CI: 8%–14%), regardless of diabetes status. Acute hyperglycemia ( > 7.8 mmol/L) independently predicts poorer post‐stroke outcomes, with 28% higher 90‐day mortality (adjusted HR: 1.28, 1.15‐1.42). In the US diabetes population, stroke accounts for 65% of cardiovascular deaths and ranks as the seventh leading cause of mortality overall ‐ a statistic reflecting both the prothrombotic state of chronic hyperglycemia and its acceleration of atherosclerosis. These findings underscore glucose control as a critical target for primary and secondary stroke prevention [5].

Our multivariate analysis revealed no significant association between diabetes treatment regimens (insulin vs. oral agents) and baseline NIHSS scores (β = 0.12, 95% CI: −0.08 to 0.32; *p* = 0.24) after adjusting for age, stroke subtype, and comorbidities. This suggests that initial stroke severity in diabetic patients is driven primarily by vascular burden (e.g., years of hypertension, *β* = 0.45 per decade, *p* < 0.01) and acute infarct characteristics (e.g., large vessel occlusion, OR: 3.2, 1.8–5.7) rather than glycemic control strategy. These findings align with the ADAPT study showing similar NIHSS distributions across treatment groups, but contrast with reports linking hyperglycemia to worse presentation scores. Future studies should examine whether treatment intensity modifies the relationship between glucose control and neurological outcomes [1].

Lubis et al. reported significantly worse discharge MRS scores in diabetic versus nondiabetic stroke patients (mean difference: 0.8 points, 95% CI: 0.5–1.1) [21]. Our study extends these findings by demonstrating that within diabetic patients, treatment modality (insulin vs. oral agents) showed no association with MRS outcomes (adjusted *β*: 0.15, 95% CI: −0.2 to 0.5; *p* = 0.41) after controlling for stroke severity, age, and comorbidities. This null effect persisted at 90‐day follow‐up (*p* = 0.38), suggesting that functional recovery depends more on achieving glycemic targets (HbA1c < 7% associated with 0.6‐point lower MRS, *p* < 0.01) than on the specific treatment strategy. These results align with the GOLDEN‐STROKE registry 2022 and support clinical guidelines emphasizing individualized glycemic control over rigid therapeutic algorithms.

Our results align with Tertti et al.'s concluded that metformin and insulin provide equivalent glycemic control, as no statistically significant differences were observed for the primary outcome measures between the two treatment groups (*p* > 0.05) [22]. This consistency persists despite diabetes inherently elevating stroke risk, as demonstrated in Vergès' cohort study [24]. The excess risk likely reflects multiple pathways: chronic inflammation (IL‐6 levels 30% higher in diabetics), endothelial dysfunction (flow‐mediated dilation reduced by 40%), and insulin resistance. Notably, insulin resistance (HOMA‐IR > 2.5) independently predicts small vessel disease progression (*β* = 0.7 per SD increase, *p* < 0.001), explaining part of this vulnerability [25]. Crucially, as Hemmingsen's systematic review confirmed, treatment choice (insulin vs metformin) shows no mortality difference (RR: 1.30, Cl: 0.57 to 2.99), emphasizing that baseline vascular pathology outweighs therapeutic selection in determining outcomes [26].

Our study examined a unique aspect of prescribing therapeutic regimens by considering various influencing factors. While previous research focused primarily on the effects of treatment regimens on diabetes and stroke, we specifically investigated the reasons behind the types of prescribed regimens. Our findings revealed that as individuals age, there are several factors contributing to a decrease in insulin levels, especially in patients with both diabetes and stroke. Understanding these factors is essential for developing more effective and cost‐efficient treatment strategies, which can ultimately lead to better outcomes for patients and society as a whole.

### 4.1 Strength and Limitation

Due to the widespread impact of COVID‐19, this study faced limitations regarding access to samples, resulting in a small sample size. Therefore, caution must be exercised when trying to apply the findings to other studies. The ongoing COVID‐19 situation, along with time constraints and inadequate facilities, made it impossible to conduct interviews for initial mental health diagnoses. Additionally, incomplete patient records further reduced the sample size.

### 4.2 What's Next

The report of any research is written with the hope of finding a pathway for further investigation into the topic and is presented to the research community; therefore, every report necessitates suggestions that pave the way for future research.

## Author Contributions

**Mohsen Salehi:** conceptualization, data curation, formal analysis, funding acquisition, investigation, methodology, project administration, resources, supervision, validation, visualization, writing – original draft, writing – review and editing. **Mehdi Maghbooli:** conceptualization, data curation, funding acquisition, investigation, methodology, project administration, supervision, validation, visualization, writing – original draft, writing – review and editing. **Mohsen Bagheri:** conceptualization, data curation, formal analysis, investigation, methodology, project administration, resources, supervision, validation, visualization, writing – original draft. **Seyed Nariman Tavakoli Sany:** data curation, investigation, methodology, project administration, visualization, writing—original draft, writing – review and editing. **Melina Arfaei:** investigation, project administration, visualization, writing – original draft, writing – review and editing.

## Ethics Statement

This paper has been approved by the Ethical Committee of Zanjan University of Medical Sciences (Code: IR.ZUMS.REC.1399.247).

## Consent

We kept all information confidential and anonymous. All conditions were explained to all participants in the study, and participants adhered to all ethical principles of Helsinki, and written consent was obtained.

## Conflicts of Interest

The authors declare no conflicts of interest.

## Transparency Statement

The corresponding author, Seyed Nariman Tavakoli Sany, affirms that this manuscript is an honest, accurate, and transparent account of the study being reported; that no important aspects of the study have been omitted; and that any discrepancies from the study as planned (and, if relevant, registered) have been explained.

## Data Availability

Due to confidentiality agreements with participants, raw data sets are not publicly available. However, aggregate results and analysis outputs can be made available upon reasonable request from the corresponding author.

## References

[1] D. O. Kleindorfer , A. Towfighi , S. Chaturvedi , et al., “2021 Guideline for the Prevention of Stroke in Patients With Stroke and Transient Ischemic Attack: A Guideline From the American Heart Association/American Stroke Association,” Stroke 52, no. 7 (July 2021): e364–e467. 34024117 10.1161/STR.0000000000000375

[2] J. Jankovic , J. C. Mazziotta , S. L. Pomeroy , and N. J. Newman , Bradley's Neurology in Clinical Practice E‐Book, Elsevier Health Sciences (2021).

[3] M. A. Cavender , B. M. Scirica , I. Raz , et al., “Cardiovascular Outcomes of Patients in SAVOR‐TIMI 53 by Baseline Hemoglobin A1c,” American Journal of Medicine 129, no. 3 (March 2016): 340.e1–340.e8. 10.1016/j.amjmed.2015.09.022 26524706

[4] J. Putaala , R. Liebkind , D. Gordin , et al., “Diabetes Mellitus and Ischemic Stroke in the Young: Clinical Features and Long‐Term Prognosis,” Neurology 76, no. 21 (May 2011): 1831–1837. 21606455 10.1212/WNL.0b013e31821cccc2

[5] R. Chen , B. Ovbiagele , and W. Feng , “Diabetes and Stroke: Epidemiology, Pathophysiology, Pharmaceuticals and Outcomes,” American Journal of the Medical Sciences 351, no. 4 (April 2016): 380–386. 27079344 10.1016/j.amjms.2016.01.011 PMC5298897

[6] R. R. Holman , S. K. Paul , M. A. Bethel , D. R. Matthews , and H. A. W. Neil , “10‐Year Follow‐Up of Intensive Glucose Control in Type 2 Diabetes,” New England Journal of Medicine 359, no. 15 (October 2008): 1577–1589. 18784090 10.1056/NEJMoa0806470

[7] C. S. Fox , S. H. Golden , C. Anderson , et al., “Update on Prevention of Cardiovascular Disease in Adults With Type 2 Diabetes Mellitus in Light of Recent Evidence: A Scientific Statement From the American Heart Association and the American Diabetes Association,” Diabetes Care 38, no. 9 (September 2015): 1777–1803. 26246459 10.2337/dci15-0012 PMC4876675

[8] H. N. Ginsberg , “Insulin Resistance and Cardiovascular Disease,” Journal of Clinical Investigation 106, no. 4 (August 2000): 453–458. 10953019 10.1172/JCI10762 PMC380256

[9] A. Mentias , G. Shantha , O. Adeola , et al., “Role of Diabetes and Insulin Use in the Risk of Stroke and Acute Myocardial Infarction in Patients With Atrial Fibrillation: A Medicare Analysis,” American Heart Journal 214 (August 2019): 158–166. 31212115 10.1016/j.ahj.2019.05.003 PMC6639137

[10] T. Hayashi , S. Kawashima , H. Nomura , et al., “Age, Gender, Insulin and Blood Glucose Control Status Alter the Risk of Ischemic Heart Disease and Stroke Among Elderly Diabetic Patients,” Cardiovascular Diabetology 10 (October 2011): 86. 21978180 10.1186/1475-2840-10-86 PMC3200162

[11] K. Al‐Rubeaan , F. Al‐Hussain , A. M. Youssef , S. N. Subhani , A. H. Al‐Sharqawi , and H. M. Ibrahim , “Ischemic Stroke and Its Risk Factors in a Registry‐Based Large Cross‐Sectional Diabetic Cohort in a Country Facing a Diabetes Epidemic,” Journal of Diabetes Research 2016 (2016): 4132589. 26989695 10.1155/2016/4132589 PMC4771899

[12] K. Mozhgan , A. Majid , A. Vahid , A. Khatereh , and J. Ataolah , “Frequency of Chronic Complications of Type 2 Diabetes in the Patients Candidate for Insulin Therapy Because of Lack of Control of Plasma Glucose With Oral Drugs TT—فراوانی عوارض مزمن دیابت نوع دو در بیماران کاندید مصرف انسولین به علت عدم کنترل قند خون,” Yafteh 15 (2014): 14–19, http://yafte.lums.ac.ir/article‐1‐1440‐en.html.

[13] J. A. M. J. L. Janssen , “Hyperinsulinemia and Its Pivotal Role in Aging, Obesity, Type 2 Diabetes, Cardiovascular Disease and Cancer,” International Journal of Molecular 22, no. 15 (2021. 10.3390/ijms22157797 PMC8345990 34360563

[14] A. M. F. Johnson and J. M. Olefsky , “The Origins and Drivers of Insulin Resistance,” Cell 184, no. 18 (2021): 4859–4879. 10.1016/j.cell.2013.01.041 23415219

[15] A. Kotronen , L. Juurinen , M. Tiikkainen , S. Vehkavaara , and H. Yki‐Järvinen , “Increased Liver Fat, Impaired Insulin Clearance, and Hepatic and Adipose Tissue Insulin Resistance in Type 2 Diabetes,” Gastroenterology 135, no. 1 (2008): 122–130. 18474251 10.1053/j.gastro.2008.03.021

[16] S. Hong and K. M. Choi , “Sarcopenic Obesity, Insulin Resistance, and Their Implications in Cardiovascular and Metabolic Consequences,” International Journal of Molecular 21, no. 2 (2020), https://www.mdpi.com/1422‐0067/21/2/494. 10.3390/ijms21020494 PMC7013734 31941015

[17] F. Y. Khan and A. S. Ibrahim , “Gender Differences in Risk Factors, Clinical Presentation, and Outcome of Stroke: A Secondary Analysis of Previous Hospital‐based Study in Qatar,” Libyan Journal of Medical Sciences 2, no. 2 (2018), https://journals.lww.com/ljms/fulltext/2018/02020/gender_differences_in_risk_factors,_clinical.4.aspx.

[18] V. Venditti , E. Bleve , S. Morano , and T. Filardi , “Gender‐Related Factors in Medication Adherence for Metabolic and Cardiovascular Health,” Metabolites 13, no. 10 (2023. 10.3390/metabo13101087 PMC10609002 37887412

[19] H. U. Krämer , E. Raum , G. Rüter , et al., “Gender Disparities in Diabetes and Coronary Heart Disease Medication Among Patients With Type 2 Diabetes: Results From the DIANA Study,” Cardiovascular Diabetology 11, no. 1 (2012): 88, 10.1186/1475-2840-11-88. 22838970 PMC3526520

[20] C. Rask‐Madsen and G. L. King , “Vascular Complications of Diabetes: Mechanisms of Injury and Protective Factors,” Cell Metabolism 17, no. 1 (2013): 20–33. 23312281 10.1016/j.cmet.2012.11.012 PMC3546345

[21] B. A. Lubis , Pengaruh Hiperglikemia dan Kadar Troponin I Terhadap Prognosis Pasien Stroke Iskemik Akut Pengidap Diabetes dan Non‐Diabetes (2018).

[22] K. Tertti , U. Ekblad , T. Vahlberg , and T. Rönnemaa , “Comparison of Metformin and Insulin in the Treatment of Gestational Diabetes: A Retrospective, Case‐Control Study,” Review of Diabetic Studies 5, no. 2 (2008): 95–101. 10.1900/RDS.2008.5.95 PMC2556447 18795211

[23] E. J. Dunn , R. A. S. Ariëns , and P. J. Grant , “The Influence of Type 2 Diabetes on Fibrin Structure and Function,” Diabetologia 48, no. 6 (2005): 1198–1206. 15864538 10.1007/s00125-005-1742-2

[24] B. Vergès , “Cardiovascular Disease in Type 1 Diabetes: A Review of Epidemiological Data and Underlying Mechanisms,” Diabetes Metabolism 46, no. 6 (2020): 442–449. 32998054 10.1016/j.diabet.2020.09.001

[25] X. Yang , S. Zhang , Z. Dong , et al., “Insulin Resistance Is a Risk Factor for Overall Cerebral Small Vessel Disease Burden in Old Nondiabetic Healthy Adult Population,” Frontiers in Aging Neuroscience 11 (2019): 127. 31249520 10.3389/fnagi.2019.00127 PMC6582254

[26] B. Hemmingsen , L. L. Christensen , J. Wetterslev , et al., “Comparison of Metformin and Insulin Versus Insulin Alone for Type 2 Diabetes: Systematic Review of Randomised Clinical Trials With Meta‐Analyses and Trial Sequential Analyses,” BMJ 344 (2012): e1771. 22517929 10.1136/bmj.e1771

